# Association between Malnutrition and Depression in Patients with Cancer: The Importance of Nutritional Status Evaluation in Cancer Care

**DOI:** 10.3390/ijerph20032295

**Published:** 2023-01-27

**Authors:** Daniele Nucci, Vincenza Gianfredi, Pietro Ferrara, Omar Enzo Santangelo, Beatrice Varotto, Alessandra Feltrin, Antonella Galiano, Mariateresa Nardi

**Affiliations:** 1 Nutritional Support Unit, Veneto Institute of Oncology IOV-IRCCS, 35128 Padua, Italy; 2 Department of Biomedical Sciences for Health, University of Milan, 20133 Milan, Italy; 3 CAPHRI Care and Public Health Research Institute, Maastricht University, 6211 Maastricht, The Netherlands; 4 Center for Public Health Research, University of Milan-Bicocca, 20900 Monza, Italy; 5 IRCCS Istituto Auxologico Italiano, 20145 Milano, Italy; 6 Regional Health Care and Social Agency of Lodi, Azienda Socio Sanitaria Territoriale di Lodi (ASST Lodi), 26900 Lodi, Italy; 7 Clinical Nutrition Unit, Department of Medicine-DIMED, University of Padova, 35128 Padua, Italy; 8 Hospital Psychology, Veneto Institute of Oncology IOV-IRCCS, 35128 Padua, Italy; 9 Department of Oncology, Oncology Unit 1, Veneto Institute of Oncology IOV-IRCCS, 35128 Padua, Italy

**Keywords:** depression, neoplasm, malnutrition, hospital anxiety and depression scale

## Abstract

Cancer patients are at risk of several comorbid conditions, including nutritional issues and mental health illnesses. The objective of the current study was to estimate the prevalence, upon hospital admission, of depression and malnutrition among adults with cancer. A retrospective chart review was conducted using health information collected as part of routine assistance. Nutritional status was measured through structured tools, including body mass index (BMI), Nutrition Risk Screening (NRS) 2002, and dietary intake needs. Depression was assessed with the Hospital Anxiety and Depression Scale (HAD). Cancer site, disease stage, length of hospitalization, age, and sex were also considered. Multivariate analyses were used to investigate the association between malnutrition and depression. In summary, our study reveals that malnutrition increases the risk of depression among cancer patients. The findings can also be used in clinical oncology for the implementation of appropriate prevention and treatment interventions in order to reduce the extent of depression and thus improve cancer patients’ quality of life and survival rate.

## 1. Introduction

In 2020, 19.3 million new cancer cases were diagnosed globally, and this is expected to reach 28.4 million cases by 2040 [1]. Thanks to an improvement in diagnosis and treatment, the survival rate has largely increased in recent decades [2]. However, the prolonging of life expectancy did not grow in parallel with quality of life both among patients and survivors [3,4,5]. Low quality of life and negative feelings may be due to several reasons, such as economic status, pain, and treatment-related side effects [6]. If those negative feelings persist for more than two weeks, depression should be suspected and diagnosed. However, despite its high burden, depression is largely underdiagnosed and untreated [7,8], particularly among patients with cancer [9].

According to a recent meta-analysis, which included 211 studies for a total of 82,426 neoplastic patients, an estimated 14% pooled prevalence of major depressive disorder (MDD) was found in those patients, also increasing to 18% when the Hospital Anxiety and Depression Scale (HAD) was used to detect these conditions [10]. Depression causes severe symptoms that negatively affect how subjects feel, think, and handle daily activities, such as sleeping, eating, or working [11]. Among others, loss of appetite is just one of the symptoms of depression that impact most on nutritional status. Malnutrition, in turn, negatively influences the response to cancer treatment [12]. In neoplastic patients, malnutrition is not only directly due to depression but can also be a tumor-related condition or be due to treatment complications (including nausea, vomiting, and loss of appetite) [12]. Moreover, malnutrition affects in a range of 25–80% of patients with cancer and is responsible for the death of almost 20% of them [13,14].

In light of the considerable burden of depression among patients with cancer and its potential association with both nutritional status and response to treatment, early identification of depression and malnutrition is extremely important. In fact, the early screening of nutritional and mental health status can help healthcare providers in understanding the nutritional and psychological support that patients may need. However, the evidence so far collected on the potential association between malnutrition and depression among neoplastic patients is scarce and not conclusive. Moreover, the factors associated with the risk of depression among hospitalized cancer patients are not completely explained.

The objective of the current study was to estimate the prevalence, upon hospital admission, of depression and malnutrition among adult cancer patients hospitalized at the Veneto Institute of Oncology. Moreover, we aimed to explore the association between malnutrition and depression and assess possible nutritional factors that may increase the risk of depression among those patients in order to highlight issues for the improvement of both activities and quality of cancer care in medical oncology.

## 2. Materials and Methods

### 2.1. Study Population, Setting and Study Design

This is a retrospective chart review conducted at the Nutritional Support Unit of the Veneto Institute of Oncology IRCCS, Padua, Italy, from May–August 2019. The Veneto Institute of Oncology is a Comprehensive Cancer Center that offers preventive, curative, and palliative treatments and improves scientific knowledge through research.

Individual data of consecutive patients admitted to the hospital were included in this study, using information collected for reasons other than research. This included the patient data of those subjects that had previously received cancer diagnosis (patients with diagnosis of cancer upon the admission day were excluded), were at least 18 years old, and were able to complete the questionnaire administered upon hospital admission. Data from patients with a history of depression, cognitive impairment, a critical condition that prevented communication with the healthcare professionals, and language or hearing issues were excluded. This study was reviewed and approved by the Ethical Committee of the Veneto Institute of Oncology IOV-IRCCS. Data included in this study were anonymized upon collection and saved in a secure database protected by a password available only to the senior resident in dietetics and clinical nutrition (M.N.). All measurements were taken during the admission day.

### 2.2. Nutritional Status Measurements

Nutritional status was evaluated through the estimation of the Body Mass Index (BMI) and the Nutrition Risk Screening (NRS) 2002. Additionally, calorie and protein intakes and weight difference between admission and discharge were also assessed. BMI provides the most frequently accepted measurement of nutritional status in adults, and it is defined by the World Health Organization (WHO) as “a person’s weight in kilograms divided by the square of the person’s height in meters (kg/m^2^)”. BMI is divided into the categories of underweight, normal, overweight, or obese, where values ranged from below 18.4, 18.5–24.9, 25.0–29.9, and ≥30.0, respectively. The body weight and height of participants were measured using a calibrated digital scale with a stadiometer (KERN MPE 250K100HM, KERN & SOHN GmbH, Balingen, Germany). Participants were asked to wear light clothes and no shoes. The body weight measurement was taken to the nearest 0.1 kg, whereas the height was measured to the nearest 0.1 cm. Body weight was also measured during the discharge day. All measurements were performed by a senior dietitian.

In addition, nutrition status was screened using the NRS-2002 developed by the ESPEN working group [15]. The NRS-2002 is based on the percentage of weight loss over the past few months, general condition, BMI, and recent food intake. It assigns a score of between 0 and 7, where 0 indicates no nutritional risk, 1–2 indicates low risk, 3–4 indicates medium risk, and a value equal to or greater than 5 indicates high risk [15]. Weight difference was determined as the difference between the patient’s weight on the admission day and his or her weight upon discharge.

To estimate the current dietary intakes, the 24-h recall method was used. This method consisted of a structured interview lasting approximately 30 min conducted by trained dietitians. During the interview, participants were asked to report detailed information (including cooking methods) about the food and drinks consumed during the previous 24 h. Dietary data were directly reported by dietitians using a nutrition analysis software, Metadieta Professional 4.3.1 (Meteda Srl, Rome, Italy), which was also used to calculate calorie and nutrient intakes.

### 2.3. Assessment of Depression

Depression was assessed by means of a validated self-administered questionnaire, the Hospital Anxiety and Depression Scale (HAD) [16]. Actually, it is based on two subscales, the HAD-A and the HAD-D, which, based on the presence and severity of symptoms, allows one to simultaneously establish both anxiety and depression, respectively. The HAD contains 14 questions, with each question being scored on a four-point scale (1 to 4), resulting in a maximum total score of 21 for anxiety and depression. A score of equal or greater than 11 on both scales is considered a significant “case” of psychological morbidity, a score of 8–10 means “borderline”, while a score of 0–7 is considered “normal”. Patients with a previous diagnosis of depressive disorders or taking antidepressants were excluded from the analysis.

### 2.4. Covariates

The cancer site, disease stage, reason for hospital admission, length of hospitalization, age, and sex were collected from medical records. Based on criteria defined by Fearon et. [17] and approved by the European Society for Clinical Nutrition and Metabolism (ESPEN) [18], cachexia was determined as disease-associated weight loss >5% during the previous 6 months or by the combination of progressive weight loss (more than 2%) and BMI < 20 kg/m^2^.

### 2.5. Statistical Analysis

All the data were elaborated using the STATA statistical software, version 14. Descriptive statistics included counts (percentages) for categorical data and mean (and standard deviation, SD) or median for continuous variables. Ordered logistic regression was performed, considering HAD-Das a dependent variable, in order to evaluate the role of the other variables. The statistical significance level chosen for all the analyses was 0.05. The model was adjusted for age and sex and the results expressed as adjusted Odds Ratio (aOR) with 95% Confidence Intervals (95% CI).

## 3. Results

### 3.1. Descriptive Characteristics of the Population

Table 1 shows the population characteristics (n = 90) stratified by depression presence according to the level of HAD-D score. The individuals had a mean age of 56.7 years (with ± 15.1 SD), and 36.7% were women. Relative percentages and means with standard deviations for several descriptive characteristics of the sample are reported in Table 1. Comparing the entire sample with subjects with missing data, the latter were mostly women, younger, and had a lower energy intake, worse disease stage, and more frequent cachexia. Supplementary Table S1 shows the prevalence by cancer site.

### 3.2. Malnutrition and Depression

Table 2 shows the association of nutritional status with depression. In the same table, we also assessed if other factors (such as disease stage, cancer site, cachexia, and HAD-A) were likely to be associated with the risk of depression among hospitalized cancer patients. Severity of malnutrition (measured through NRS-2002), disease stage, cachexia, and anxiety were associated with the increased risk of depression in a statistically significant manner. In particular, every unit increase of NRS-2002 was associated with a 71% higher risk of depression. Significant higher odds of depression were also found according to disease stage (aOR2.04; 95% CI 1.34–3.11), cachexia (aOR3.18; 95% CI 1.33–7.60), and anxiety score measured through HAD-A score (aOR1.71; 95% CI 1.44–2.03).

## 4. Discussion

The aim of this observational study was to collect data on prevalence of malnutrition and depression in hospitalized cancer patients, offering an interesting insight into the association between these conditions. Regarding nutritional status, our results found that 60% of the cancer patients had a normal BMI. However, more informative than body weight or BMI was the NRS-2002, which is widely used to assess the nutrition of patients at risk for malnutrition. In fact, the nutritional assessment performed using this tool revealed that more than one third of the sample (34.5%) was at medium-high risk of malnutrition. The percentage increased when patients with depression were considered (50% of patients with depression scored high in NRS). However, only one patient was classified as having a high risk of malnutrition. Notably, cachexia was present in 38.9% of the patients. On the contrary, a low risk of malnutrition was detected in approximately 40% of the borderline/depressed patients.

Moreover, more than a quarter of patients had an abnormal/borderline HAD-D. In particular, the prevalence found is in line with evidence so far available that has documented an average rate of depression in around 20% of patients with cancer, also peaking up to more than 50% [19,20].

We also created logistical regression models and found that the increase in NRS-2002 score was associated with the HAD-D score, and thus the level of depression. Furthermore, the cancer site (i.e., gastrointestinal cancer), an advanced stage of disease, cachexia, and increasing HAD-A were found to increase the odds for depression occurrence according to the HAD-D scale.

The diagnosis of cancer directly affects patients’ lives, leading to a number of lifestyle changes, which may be the source of considerable psychological and emotional stress [21]. Depression is thus a common comorbidity in medical oncology, and the lack of recognition and diagnosis negatively impacts patients’ quality of life and survival [19,22]. Some drivers have been described, such as the site and prognosis of primary cancer (for instance, it is most common in pancreatic cancer, probably due to its poor prognosis) [23], the presence of metastases and cancer pain, female gender; other possible factors are likely to be related to cancer-attributable changes in lifestyle and habits towards those mostly correlated with incident depression [24,25,26]. In general, all these trigger a 3-fold prevalence of depression in these patients compared to the general population [27].

Several pieces of research have evidenced associations between dietary patterns and depression [28,29]. Malnutrition, in particular, has shown a direct independent association with depression, both in the general population and in specific groups [30,31]. With progression of the disease, cancer patients developed weight and BMI loss, with changes in dietary intakes of most nutrients, which result in energy intake deficiency, altered anthropometric indices, and poor nutritional status [32,33,34]. Our analysis observed a high significance between all the nutritional indices and scores explored and the HAD-D score, finding that nutrition was directly correlated with depression in cancer patients. Similarly, higher odds for depressive status were also seen in patients at greater risk of cancer-related malnutrition, such as those with gastrointestinal cancers, progressive disease stages, and cachexia [14,34,35].

The mechanisms by which nutritional status increases the rate of depression among cancer patients deserve further research [31], but our analysis captured important independent predictors, which may help to provide a better understanding of the drivers of nutrition-related depression in medical oncology. In fact, data from our study add important knowledge to the existing body of evidence on this research topic. They mirrored the prevalence of nutritional risk, anxiety, and depression determined by a Canadian study on the nutritional and psychosocial status of patients with colorectal cancer, which also emphasized the need to use nutritional and psychosocial screening tools in oncology settings as confirmed by the analysis presented [35]. Again, in a cohort of older men with advanced prostate cancer, depression and nutrition risks were suggested [36]. Lastly, it is also worth mentioning that Van Liew et al. observed, among head and neck cancer patients, that nutritional deficit and weight loss incrementally worsen to the extent that depressive symptoms increased (and vice-versa) [37].

In brief, it is important to identify early nutritional deficit in cancer patients in the context of its relationship with depression and its adverse outcomes in order to improve patients’ health and poor quality of life [29,38]. Cancer patients experience modifications in diet and dietary habits because of the disease and treatments, and some changes are also prompted as part of tumor therapies [39,40,41]. In this sense, it is worth noting that measures of nutritional status of patients with cancer is not routinely performed in standard oncology care: according to the found evidence, around 50% of malnourished cancer patients do not undergo nutritional screening as part of their care [42,43,44]. This has been also attributed to a low level of training of non-nutritionist doctors regarding action protocols to be implemented, although evidence demonstrated that educational initiatives targeting clinicians effectively change their behaviors towards nutritional risk assessments [14,45]. Indeed, expert nutritionists play an essential role in the evaluation of nutritional status in oncology in order to both prevent malnutrition and its consequences. Actually, introducing nutritional screening in routinary cancer care is important to detect patients at higher risk of malnutrition and complications, as depression. This also concerns the possibility of avoiding a significant impact on health care and systems, averting disease complications and resource consumption. Therefore, our findings might help clinicians to implement the most appropriate interventions and inform policymakers providing actionable evidence-based metrics which are essential to enhance cancer care [40,46,47,48]. Furthermore, highlighting the association of depression with malnutrition, results from the presented study also remarked the clinical significance of a complete assessment of patients’ mental health. Studies found that this is not easy on clinical grounds and this difficulty is reflected into the heterogeneity of depression rates in cancer patients, which vary from 5 to 60% [49]. Comprehensive training programs for clinicians should therefore address this point.

Lastly, it is worth considering the above in the context of the impact that the coronavirus disease 2019 (COVID-19) pandemic has had on health and healthcare [50,51]. It caught healthcare systems off guard worldwide, with rapid changes in routine care and assistance of patients, with significant negative effects on their health, including worsening of the mental health burden [52,53]. In fact, the dynamics of COVID-19 prompts the need to implement further coordination of care as well as transitional care interventions for the care of the most vulnerable populations (such as cancer patients), also through the use of health technology and digital health tools [48,50,51,54,55], which could provide correct and updated follow-up information on nutritional and psychological statuses in cancer patients.

### Limitations and Strengths

The limitations of our study include the fact that the number of hospital cancer patients who were included in the analysis was limited as a real-world study; yet, this number is roughly in line with the sample size of existing similar literature [35,36]. This reflects the difficulty and lack of comprehensive nutritional and psychological screenings in oncology settings. Also, due to the utilization of data collected for reasons other than research, it was not possible to assess other possible factors that could have had an impact on depression prevalence and severity. For instance, the mineral and vitamins deficiency are not routinely investigated, and consequently, we could not assess the potential association. Similarly, the socioeconomic statuses of the patients were not taken into account.

Despite these limitations, as the participants in this study represented the standard population who are admitted to cancer units, it is possible to state that the selected cohort is representative of the target population. Moreover, the enrollment of a consecutive hospital cohort reduces problems of selection and participant bias. We also used data based on validated diagnostic instruments. In fact, NRS 2002 is considered the most suitable tool for assessing nutritional status in cancer patients, which is designed to include measurements of potential current malnutrition and disease severity [42]. The HAD scale, in turn, is valid for the measurement of psychological morbidity in cancer patients and provides clinically meaningful results as a psychological screening tool for clinical groups and studies that wish to consider multiple aspects of illness and quality of life [56,57]. It is sensitive to changes both in the course of the disease and in response to medical and psychological interventions [58].

## 5. Conclusions

In summary, our study reveals that one third of adult cancer patients scored high in NRS 2002 (indicating a malnutrition status) and that poor nutritional status was associated with depression. Many of the investigated factors are both preventable and treatable through existing interventions. The evidence of an association between malnutrition and depression confirms the importance of systematic assessments of the nutritional and mental health characteristics of those patients. Findings from this study may thus help clinicians and nutritionists to implement the most appropriate prevention and treatment interventions in order to reduce the extent of depression and thus improve cancer patients’ quality of life and survival rate.

## Figures and Tables

**Table 1:** Descriptive characteristics of the sample.

	Depression (Based on HAD-D score)N (%)				
Variable	Normal	Borderline	Abnormal	Missing	Total
Sex					
Women	15 (26.8)	9 (64.3)	1 (16.7)	8 (57.1)	33 (36.7)
Men	41 (73.2)	5 (35.7)	5 (83.3)	6 (42.9)	57 (63.3)
Age *	55.8 ± 15.6	62.1 ± 11.8	57.0 ± 12.4	54.4 ± 17.2	56.7 ± 15.1
Disease stage					
I	8 (14.3)	0	0	1 (7.1)	9 (10.0)
II	9 (16.1)	0	0	1 (7.1)	10 (11.1)
III	10 (17.9)	0	0	1 (7.1)	11 (12.2)
IV	29 (51.7)	14 (100)	6 (100)	11 (78.7)	60 (66.7)
Cancer site					
Gastrointestinal	21 (37.5)	11 (78.6)	4 (66.7)	6 (42.9)	42 (46.7)
Others	35 (62.5)	3 (21.4)	3 (33.3)	8 (57.1)	48 (53.3)
Weight (in kg) *	72.3 ± 13.5	65.4 ± 12.6	62.5 ± 10.8	63.5 ± 13.4	69.2 ± 13.6
Weight difference upon discharge *	0.22 ± 1.2Median = 0	0.42 ± 2.3Median = 0	0 ± 2.3Median = −1	0.2 ± 0.0Median = 0	0.2 ± 2.0Median = 0
Body mass index *	24.2 ± 4.0	24.2 ± 4.9	20.1 ± 2.3	23.3 ± 3.8	23.8 ± 4.1
Underweight (<18.5)	1 (1.8)	1 (7.1)	1 (16.7)	2 (14.3)	5 (5.6)
Normal (18.5–24.9)	32 (57.1)	8 (57.1)	5 (83.3)	9 (64.3)	54 (60.0)
Overweight (25.0–29.9)	19 (33.9)	3 (21.4)	0	3 (21.4)	25 (27.8)
Obese (≥30.0)	4 (7.1)	2 (14.3)	0	0	6 (6.7)
Daily energy intake *	1504.8 ± 252.4	1483.3 ± 469.7	1420.0 ± 472.5	1250.0 ± 212.1	1465.9 ± 212.1
Cachexia					
Yes	16 (28.6)	8 (57.1)	4 (66.7)	7 (50.0)	35 (38.9)
No	40 (71.4)	6 (42.9)	2 (33.3)	7 (50.0)	55 (61.1)
NRS-2002 *	1.8 ± 1.0	3 ± 1.4	2.8 ± 1.3	2.1 ± 1.1	2.1 ± 1.3
1	32 (57.1)	3 (21.4)	1 (16.7)	6 (42.9)	42 (46.7)
2	10 (17.9)	2 (14.3)	2 (33.3)	3 (21.4)	17 (18.9)
3	9 (16.1)	2 (14.3)	0	3 (21.4)	14 (15.6)
4	5 (8.9)	6 (42.9)	3 (50.0)	2 (14.3)	16 (17.8)
5	0	1 (7.1)	0	0	1 (1.1)
HAD *	8.7 ± 3.9	18.1 ± 2.1	21.7 ± 5.6	n.a.	11.3 ± 6.2
HAD-A *	5.0 ± 2.5	8.9 ± 1.7	8.5 ± 5.0	n.a.	6.0 ± 3.2
Normal (0–7)	47 (83.9)	4 (28.6)	3 (50.0)	n.a.	54 (60.0)
Borderline (8–10)	7 (12.5)	5 (35.7)	1 (16.7)	n.a.	13 (14.4)
Abnormal (11–21)	2 (3.6)	5 (35.7)	2 (33.3)	n.a.	9 (10.0)
Missing	n.a.	n.a.	n.a.	14 (100)	14 (15.6)

* Summarized by mean and standard deviation (SD). Abbreviations: HAD, Hospital Anxiety and Depression Scale; HAD-D, HAD scale for depression; HAD-A, HAD scale for anxiety; NRS-2002, Nutrition Risk Screening 2002.

**Table 2:** Ordered logistic regression. The odds ratio adjusted for age and sex are presented.

Dependent Variable: HAD-D				
Independent Variable		aOR	95% CI	*p*-Value
NRS-2002	as the unit increases	1.71	1.20–2.43	0.003
BMI	as the unit increases	0.96	0.87–1.06	0.462
Daily energy intake	as the unit increases	0.99	0.99–1.00	0.710
Daily protein intake	as the unit increases	1.02	0.98–1.06	0.398
Weight difference	as the unit increases	0.98	0.82–1.20	0.900
Disease stage	as the unit increases	2.04	1.34–3.11	0.001
Disease stage				
I		Ref.	-	-
II		0.83	0.16–1.21	0.823
III		1.14	0.22–5.97	0.878
IV		5.90	1.50–23.15	0.011
Cancer site				
Gastrointestinal cancer		Ref.	-	-
Others		0.25	0.10–0.60	0.002
Cachexia	as the unit increases	3.18	1.33–7.60	0.009
HAD-A	as the unit increases	1.71	1.44–2.03	<0.001

Abbreviations: HAD, Hospital Anxiety and Depression Scale; HAD-D, HAD scale for depression; HAD-A, HAD scale for anxiety; NRS-2002, Nutrition Risk Screening 2002; BMI, body mass index; aOR, adjusted odds ratio; 95% CI, 95% confidence interval; Ref., reference category.

## Data Availability

All data are available in the current publication.

## Supplementary Materials

The following supporting information can be downloaded at: https://www.mdpi.com/article/10.3390/ijerph20032295/s1, Table S1: Prevalence by cancer site.
