# Supplementary material for: Association between Malnutrition and Depression in Patients with Cancer: The Importance of Nutritional Status Evaluation in Cancer Care
Source: Int J Environ Res Public Health. 2023 Jan 27;20(3):2295. doi: 10.3390/ijerph20032295 (PMC9916136; doi:10.3390/ijerph20032295)
Supplement: Supplementary file 1 [file ijerph-20-02295-s001.zip › Supplementary Table S1.pdf]

Supplementary Table S1. Prevalence of depression by cancer site.

**Cancer site**	**N (%)**	
Colorectal cancer	22 (24.4)	
Soft tissue cancer	15 (16.7)	
Non-Hodgkin's lymphoma	13 (14.4)	
Pancreatic cancer and bile ducts cancer	10 (11.1)	
Gastric cancer	7 (7.8)	
Bone cancer	6 (6.7)	
Testicle cancer	4 (4.4)	
Esophageal cancer	3 (3.3)	
Renal cancer	2 (2.2)	
Hepatic cancer	2 (2.2)	
Unspecified primary cancer	2 (2.2)	
Meningeal cancer	1 (1.1)	
Penile cancer	1 (1.1)	
Lung cancer	1 (1.1)	
Adrenal cancer	1 (1.1)	
